# Cell Transdifferentiation and Reprogramming in Disease Modeling: Insights into the Neuronal and Cardiac Disease Models and Current Translational Strategies

**DOI:** 10.3390/cells10102558

**Published:** 2021-09-27

**Authors:** Rajkumar Singh Kalra, Jaspreet Kaur Dhanjal, Mriganko Das, Birbal Singh, Rajesh Naithani

**Affiliations:** 1 AIST-INDIA DAILAB, National Institute of Advanced Industrial Science & Technology (AIST), Higashi 1-1-1, Tsukuba 305-8565, Japan; rajkumar-singh@oist.jp; 2 Department of Computational Biology, Indraprastha Institute of Information Technology Delhi (IIITD), Okhla Industrial Estate, New Delhi 110020, India; 3 Department of Biotechnology, Vel Tech Rangarajan Dr. Sagunthala R&D Institute of Science and Technology, Chennai 600062, India; dasmriganko@gmail.com; 4 ICAR-Indian Veterinary Research Institute, Regional Station, Palampur 176061, India; bsbpalampur@yahoo.co.in; 5 Himalayiya University, Dehradun, Uttarakhand 248001, India; rajesh.naithani@gmail.com

**Keywords:** transdifferentiation, cell reprogramming, induced pluripotency, disease modeling, neuronal diseases, cardiac disease, regenerative medicine, therapeutic strategies

## Abstract

Cell transdifferentiation and reprogramming approaches in recent times have enabled the manipulation of cell fate by enrolling exogenous/artificial controls. The chemical/small molecule and regulatory components of transcription machinery serve as potential tools to execute cell transdifferentiation and have thereby uncovered new avenues for disease modeling and drug discovery. At the advanced stage, one can believe these methods can pave the way to develop efficient and sensitive gene therapy and regenerative medicine approaches. As we are beginning to learn about the utility of cell transdifferentiation and reprogramming, speculations about its applications in translational therapeutics are being largely anticipated. Although clinicians and researchers are endeavoring to scale these processes, we lack a comprehensive understanding of their mechanism(s), and the promises these offer for targeted and personalized therapeutics are scarce. In the present report, we endeavored to provide a detailed review of the original concept, methods and modalities enrolled in the field of cellular transdifferentiation and reprogramming. A special focus is given to the neuronal and cardiac systems/diseases towards scaling their utility in disease modeling and drug discovery.

## 1. Introduction

**Figure 1:**
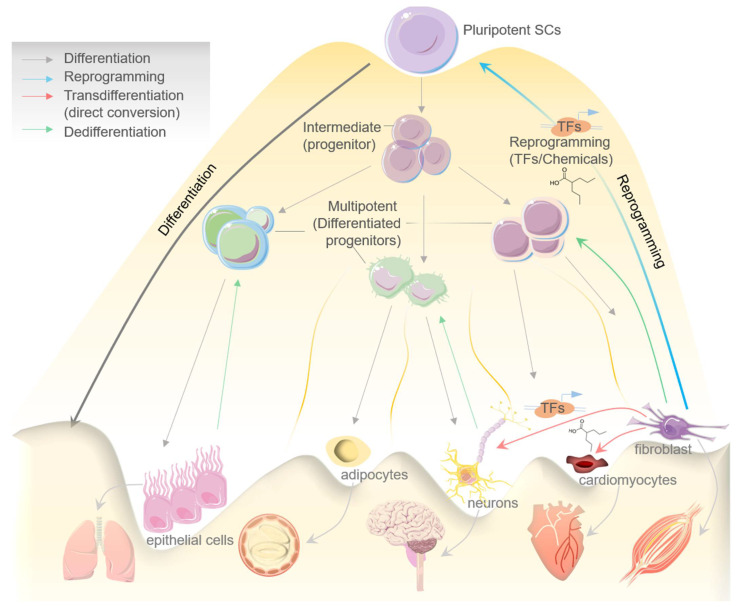
Schematic diagram showing processes of differentiation, reprogramming, transdifferentiation (direct conversion), and dedifferentiation in determining the cells fate, while dedifferentiation represents a reverse step in this process. Model exhibiting the pluripotent stem cells differentiation to intermediate progenitor SCs, then to multipotent SCs/differentiated progenitors, and eventually to mature tissue-specific specialized cells. Reprogramming indicates reverting back a mature cell into induced pluripotent stem cells (may consist of an intermediate step/cell population) with the help of specific TFs and chemicals/small molecules. Transdifferentiation represents the direct conversion of a specialized mature cell into another cell type by the help of specific TFs and chemicals/small molecules.

The quenching of cell stemness as cell progressively proliferates and acquires a differentiated state was initially thought to be an irreversible mechanism [1]. The canonical design of a biological process such as cell differentiation has now largely been disproved in the light of emerging evidence about the cellular reprogramming and transdifferentiation mechanisms that potentiate conversion of a lineage-specific, differentiated cell into another/different lineage/cell type [2]. In the process of differentiation, a pluripotent stem cell systematically proliferates and undergoes the intermediate/progenitor and differentiated progenitor/multipotent stages before losing its plasticity and dividing terminally into the specialized/mature cells which constitute an organ or tissue [3]. Mechanistically, when a differentiated cell reverts to its parental lineage or less-differentiated cell to acquire a proliferative phenotype, the process is generally known as dedifferentiation, while transdifferentiation suggests the direct conversion of a differentiated cell type to another differentiated cell type without entering a pluripotent state. Therefore, transdifferentiation is often called direct cell reprogramming [3,4]. Both differentiation and transdifferentiation events can occur naturally [2]. In contrast, the process of cell reprogramming or induced pluripotency, which refers to the process of reverting specialized/differentiated cells to the induced pluripotent stem cells (iPSCs) state, is largely artificial [5] (Figure 1).

Cell reprogramming can be induced artificially by chemicals/small molecules or by expressing certain transcription factors (TFs), which reprogram a cell to enter an intermediate or pluripotent state [6] (Figure 1). Davies and Weintraub, in the earliest report in 1987, firstly demonstrated the ability of lineage-specific TFs to govern cell fate [7]. Murry et al., later in 1996, brilliantly showed that MyoD expression across different cell lines in vitro stimulates muscle-specific genes’ expression and may further convert these cells into myoblasts [8]. Accumulating evidence in the last three decades has significantly established cellular reprogramming and transdifferentiation in mammals; however, events altering cell fate were also seen to occur naturally [9].

The pathological side of these processes is known in clinical practice, for instance in Barret’s metaplasia, Cdx2 activation transdifferentiates stratified squamous cells into epithelial cells, which potentiates esophagus carcinoma [10]. Earlier reports showed that the transdifferentiation of diverse cell types into myofibroblasts may cause fibrosis in the case of injury or chronic damage to the liver [11], kidney [12], and muscle [13], while a natural transdifferentiation mechanism can be seen in heart [14], liver [15], and in the lens regeneration process in axolotls [16]. Although such remarkable regeneration abilities produced by endogenous transdifferentiation are largely restricted to lower vertebrates, mammals exhibit limited features [9]. For instance, after injury, Lgr5+ led transdifferentiation induces the revival of the hair follicular cells in the inner ear, a rare feature that is exclusive to the neonatal stages. However, in the adults it fails to repair injury significantly [17].

Developments in this field have largely been fueled by investigations into these model organisms and their regenerative abilities, and from the accumulating knowledge on small molecules/chemicals and key cell fate-regulators. The latter includes key transcription factors that can instigate cellular reprogramming and transdifferentiation [18,19,20,21,22], which is largely seen as a promising therapeutic strategy in disease modeling [5,23,24,25,26]. In the following section, we review the role of diverse factors involved in cellular transdifferentiation towards regulating the cell fate in disease modeling.

## 2. Cell Transdifferentiation: An Overview

A recent development in transdifferentiation or direct lineage-reprogramming— where a cell converts into another cell type without crossing the pluripotent state—offered novel applications to produce functional cells/tissues in disease modeling [18]. Although several functional cell types, including cardiomyocytes, neurons, progenitor/stem cells, hepatic stem cells, hepatocytes, and blood/hematopoietic stem cells have been obtained from fibroblasts/other somatic cells in vitro using the TFs or chemical-mediated transdifferentiation approach, a greater focus of translational research on neural and cardiac cells has been evident.

### 2.1. TFs-Mediated Transdifferentiation and Scope in Disease Modeling

An increasing therapeutic focus in neural and cardiac research prompted a series of investigations in the area and led to significant progress being made in the field of in vitro and in vivo transdifferentiation. Multiple studies produced transdifferentiated cells that phenotypically resemble their natural counterparts and maintain prolonged functionality within the neural and cardiac tissues (Table 1).

#### 2.1.1. TFs-Mediated Neural Stem/Progenitor Cell Transdifferentiation

**Figure 2:**
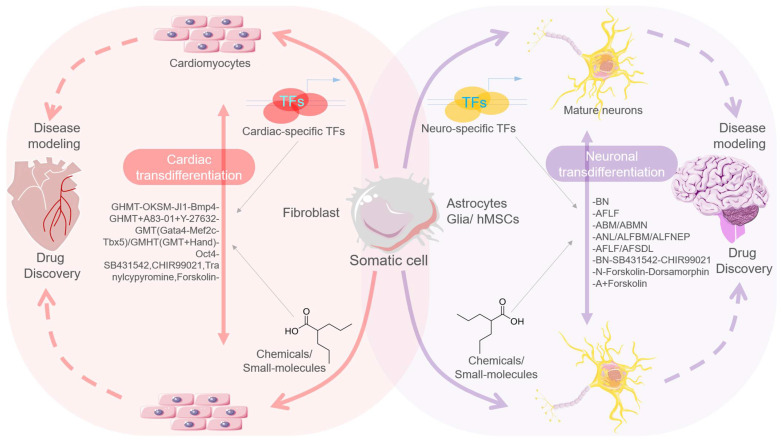
Schematic diagram showing processes of cardiac and neuronal transdifferentiation from a normal somatic cell. Transdifferentiation of somatic cell/fibroblast into cardiomyocytes is induced by certain combinations (listed) of cardiac-specific TFs or chemicals/small molecules (**left side**); while transdifferentiation of somatic cells, specifically of astrocytes, glia, or human mesenchymal stem cells (hMSCs), into mature neurons can be induced by certain combinations (listed) of cardiac-specific TFs or chemicals/small molecules (**right side**). Both processes highlight translational and regenerative potential of transdifferentiation in disease modeling and drug discovery.

A high prevalence of neurodegenerative diseases encouraged transdifferentiation strategies to regenerate neurons/neural networks in the central nervous system (CNS) and brain. By instigating in vivo transdifferentiation in the brain of an adult mouse, a report in 2013 first demonstrated a proof-of-principle of the mechanism. In this report, Torper and colleagues enrolled both resident cells, i.e., astrocytes, and transplanted cells, i.e., fibroblasts and astrocytes, as carrier cells, and performed delivery of *Ascl1*, *Myt1l*, and *Brn2a* TFs using a viral vector [27]. The adopted transgenic and transplantation approach comprehensively demonstrated ease at yielding specialized populations, including dopaminergic, and DA neurons. They suggested the suitability of astrocytes for neuron transdifferentiation considering their enriched and ubiquitous presence in the brain [27] (Figure 2).

In another report, Niu et al. adopted a lentiviral-based astrocyte-specific GFAP promoter system and transdifferentiated the target population into neuroblast cells in the mouse brain [28]. Of note, they found that a single TF-*Sox2* was sufficient to transdifferentiate the target cells. Importantly, this strategy produced proliferative precursor cells, such as native neuroblasts, and called them induced adult neuroblasts (iANBs). They enrolled BNTP and Noggin—neurogenic factors and VPA, VP—epigenetic modulator to further support iANBs maturation towards developing into electrophysiologically efficient, circuit-integrated mature neurons in vivo. Such iANBs–derived mature neurons were found to be associated with different subtypes yet mainly expressed calretinin [29]. They later carried out *Sox2*-mediated transdifferentiation of astrocytes to neuroblasts in spinal cord injury in another report [30]. In the case of spinal cord injury, the GFAP-lentiviral delivery system was seen to improve astrocyte proliferation in the region and to form a glial scar, yet it was later found to restrict axon regeneration [31]. However, in the in vivo systems, transdifferentiation efficiency in the adult brain was limited, as only 3–6% of astrocytes were transdifferentiated into iANBs. Additionally, iANB-derived GABAergic neurons were seen to form synapses with the native neuronal networks; however, their electrophysiological function could not be confirmed. Moreover, the extent of viral load as a vector was suggested to be disadvantageous for transdifferentiation efficiency, and the survival of transduced cells was also found to be a limitation towards scaling its utility for disease modeling (Table 1).

Taking two brain-injury cases—including stab-injured brain and Alzheimer’s disease (AD) brain tissue—another report enrolling *NeuroD1* showed an efficient transdifferentiation of glia/astrocytes and NG2 cells into the functional and mature neurons in mouse brain. These mature neurons were locally integrated into the neural circuits and sustained survival for about 2 months [32]. It was promising to see that *NeuroD1* has the potential to transdifferentiate astrocytes into the glutamatergic neurons in the human in vitro model; however, no data on behavioral rehabilitation were captured.

Moreover, the post-mitotic status of neurons makes it tougher to systematically acquire transdifferentiation in target cells. Rouaux and Arlotta, by taking *Fefz2*, i.e., a key corticofugal projection factor, used the utero electroporation method and performed transdifferentiation of the excitatory neurons in the cortex region of mouse brain [33]. They affirmed that a significant population of transdifferentiated cells retained these changes for about 4 weeks. Simultaneously, another report demonstrated similar results. In this report, De la Rossa and colleagues introduced *Fefz2* in the brain cortex, yet they used the iontoporation and CreERT2 methods to stimulate the TF expression at different developmental stages [34]. Using specific promoters, they controlled TF expression in specific populations and effectively transdifferentiated L4 spiny neurons into L5B neurons that exhibited native phenotype, transcriptomic signature, axon character, and bidirectional signal connectivity (Table 1).

Recently, Qin et al. demonstrated the transdifferentiation of human fibroblast cells into DA-neuron-like cells by using a combination of protein factors and small molecules [35]. Their method exhibited efficient direct conversion, as 95% of yielded cells were TUJ1-positive, and the process did not include an intermediate neural stem/progenitor stage. In another recent report, Song et al., by using a doxycycline-inducible TFs system (carrying Ngn2, Ascl1, and Dlx2) in human pluripotent stem cells, performed the successful transdifferentiation of these cells into excitatory and inhibitory neurons, exhibiting an equivalent phenotype and molecular signature [36].

Although these studies still do not qualify directly for therapeutic applications or diseases modeling, they do demonstrate proof-of-principal that neurons with post-mitotic state can be transdifferentiated, and cell-to-cell conversion can be programmed. These reports decisively affirmed that TFs-mediated neural stem/progenitor cells’ transdifferentiation can critically shape its therapeutic applications, and utility in disease modeling, more specifically in neurodegenerative and age-related neuronal diseases.

#### 2.1.2. TFs-Mediated Cardiomyocytes’ Transdifferentiation

Given the clinical prevalence and translational significance of cardiovascular disease [44], the scope of somatic cell transdifferentiation to cardiomyocytes (CMs) has been widely investigated. In one of the earliest reports, the researchers tried to revive an injured rat heart by instigating MyoD-mediated transdifferentiation of the heart fibroblasts into skeletal muscle cells [8]. Although the study involved the transfection of MyoD-expressing cells and embryonic-MHC, no data on the cell fusion or maturation (embryonic myofibroblasts into functional cells) and their integration with native cardiomyocytes were obtained. However, the presence of myofiber like cells in the cardiac microenvironment hinted at an incomplete maturation of the cells into a skeletal muscle phenotype. Furthermore, an elicited immune response against the adenoviral vector that was used in high-dose in the study was suggested to compromise the outcome (Figure 2). This earliest report in many ways advanced the field by providing initial experimental knowledge and by facilitating further attempts that enrolled multiple TFs that aimed to regenerate and obtain mature CMs. Consistent with this, two independent reports in 2012 adopted a similar strategy and endeavored to regenerate the myocardium post-myocardial infarction (MI) by transdifferentiating the heart fibroblasts into induced-CMs (iCMs) in mouse heart. Taking the *Gata4*, *Mef2c,* and *Tbx5* (GMT) transcription factors, Inagawa et al. and Qian et al. utilized a retroviral system to selectively transfect dividing cells to deliver it to the cardiac fibroblasts exclusively, avoiding post-mitotic CMs [37,38]. Qian and colleagues further validated the fibroblastic origin of iCMs, and the transdifferentiated cells shared similar features to native cells in terms of morphology, transcriptome, sarcomeric design, and electrical behavior. The iCMs acquired identical electrical stimulation function to ventricular CMs and significantly improved cardiac function, while no arrhythmias or cardiac death events were reported. By estimate, the adopted strategy was found to transduce 4% of the fibroblasts in infarcted areas, out of which only 10–15% were found to be transdifferentiated, thus hinting at lesser transdifferentiation efficiency. This was even more evident in the investigation by Inagawa et al. where resulting iCMs largely lacked maturation. Christoforou and colleagues further showed that expression of *Myocd* and *Srf*—by itself or in combination with *Smarcd3* and *Mesp1*—can enhance the basal yet needed cardio-inducing impact of the known TFs *viz. Gatat4, Ata4, Tbx5,* and *Mef2c* during direct cellular reprogramming [45]. Later, in 2017, by using a consortium of TFs (*Gata4, Tbx5, Mef2c, Myocd, Nkx2-5*) and microRNAs (miR-1 and miR-133a) they further successfully achieved the reprogramming of human dermal fibroblasts into iCM-like cells. Of note, enriched expression of cardiac-specific genes, activated myocardial and physiology-related pathways, and decreased levels of fibroblastic markers were evidently seen in these reprogrammed iCM-like cells [46].

Given the in vivo scenario, it was believed that transdifferentiation achieves better results in vivo than in in vitro culture, probably due to a more native/homogenous microenvironment that effectively sustained cell survival and maturation [37,47]. These reports emphasized the role of the native microenvironment in achieving functionally mature transdifferentiated cells, which could enable the revival of injured tissue. Adopting a similar strategy and an additional TF-*Hand2*, Song et al. further aimed to improve the transdifferentiation efficiency [39]. This strategy indeed improved the iCM generation efficiency to 6.8% and also the cardiac function recovery after MI.

Moreover, another study also endeavored to achieve cell transdifferentiation after MI; however, it adopted a lentiviral approach including a cocktail of four micro-RNAs *viz.* miRNA 1, 133, 208, and 499 that have functions in CM development [41]. Another report from this group revealed that the electrical features of transdifferentiated iCMs subsets were comparable to the endogenous CMs, which was reflected in the moderately improved cardiac function [40]. However, a lesser efficiency of transdifferentiation and immature iCMs hinted at the limitations of the adopted strategy and stressed the need for process optimization. Further, Mathison et al., in 2017, using an adenoviral vector system expressing GMT cardiac TFs, directly transdifferentiated the fibroblast cells into cardiomyocytes [48]. These cells were found to improve cardiac function in the post-infarct rat hearts. These reports demonstrated effective transdifferentiation as affirmed by lineage tracing or adopted co-transduction strategies, and by validation of iCMs fibroblastic origin. However, it was believed that transdifferentiation efficiency could be improved further if this step were delayed after injury, as it could allow more fibroblasts migration to the infarct site [8] (Table 1).

### 2.2. Chemicals/Small Molecule-Mediated Transdifferentiation

Growing concerns on the safety of viral DNA integration (delivery vehicle/plasmid used in TFs-mediated transdifferentiation) and stochastic risk of oncogene potentiation, led many researchers to explore the utility of chemical/compounds or small molecules with a revised transdifferentiation efficiency. In this section, we review diverse approaches involving chemicals/small molecules for neural stem/progenitor and cardiac cells’ transdifferentiation (Table 1).

#### 2.2.1. Chemical/Small Molecule-Mediated Neural Stem/Progenitor Cell Transdifferentiation

Cheng et al., in 2014, were the first to use human urinary and mouse fibroblast cells and demonstrate the formation of fully chemical-induced neural progenitor cells (ciNPCs) by three small molecule cocktails that included CHIR99021 (GSK3 inhibitor), VPA (HDAC inhibitor), and Repsox (TGFβ inhibitor). They achieved the generation of ciNPCs under physiological hypoxic (5% O_2_) condition, using no expression of any exogenous TFs/genes [49]. An alternative cocktail including LiCl, NaB, and SB431542 or Li_2_CO_3_, TSA, and Tranilast small molecules exhibited similar efficacies of ciNPC generation when enrolled with inhibitors of HDAC, GSK3, and TGFβ signaling. These ciNPC exhibited multipotent phenotypes and efficiently differentiated into different neural cell types in both in vitro and in vivo systems (Figure 2).

Similarly, using a cocktail of nine small molecules (M9), Zhang et al. further efficiently induced transdifferentiation into mice fibroblasts to produce neural stem cell-like cells (ciNSLCs) [50]. Initially, they enrolled three small molecules (LDN193189, A83-01, CHIR99021) and one growth factor (basic fibroblasts growth factor; bFGF) known to modulate epigenetic and neuro-generative signaling. On top of these three molecules, the other small molecules were assessed. Of note, Hh-Ag 1.5 and retinoic acid distinctly enriched Sox2^+^/Nestin^+^ cells and therefore these two chemicals were again included in the basal conditions to screen small molecules along with six chemicals. Interestingly, three chemicals, *viz.* Parnate, RG108, and SMER28, were further shown to improve the induction of Sox2^+^/Nestin^+^ cells, thereby establishing the M9 cocktail. They also highlighted the role of the Elk1 and Gli2 transcription factors, which function in MP-induced signaling and activate key neural genes that have specialized functions in determining the neural identity. Cortes-Medina et al., in 2019, by using a cocktail of I-BET151, forskolin, CHIR99021, Y-27632, RepSox, and dbcAMP small molecules, demonstrated neural transdifferentiation potential of human mesenchymal stem cells [51]. They also revealed that the addition of neurotrophic factors to the cocktail further increased the cells’ numbers significantly, and the cells exhibited a neuronlike morphology and the expression of neural markers (Table 2 and Table 3).

#### 2.2.2. Chemical/Small Molecule-Mediated Neuronal Transdifferentiation

Using the full-chemical strategy, in 2015, two simultaneous reports successfully produced human and mouse neurons from their fibroblast cells [52,53]. Using *Ascl1*, i.e., a regulator of neuronal cell fate, Li et al. constituted a neural transdifferentiation system and screened small molecules that led to the finding of four molecules, *viz*. Forskolin, CHIR99021, ISX9, and SB431542, which promoted the *Ascl1*-mediated generation of neuronal cells from mouse fibroblasts [52]. Of note, a cocktail of four molecules was found to efficiently stimulate neuronal transdifferentiation and it was evident without *Ascl1*. They also identified an additional small molecule *viz.* I-BET151 that strikingly improved the transdifferentiation efficiency (>90% TUJ1^+^). Later, since SB431542 activity was found to be negligible, the final cocktail constituted Forskolin, CHIR99021, ISX9, and I-BET151. On the mechanistic side, they elucidated that I-BET151, a BET family bromodomain inhibitor essentially suppresses fibroblast-specific genes, whereas ISX9, a neurogenesis inducer, activates neuron-specific transcriptional program [52]. In another report, Hu et al. tested CHIR99021, VPA, and Repsox (VCR) small molecules (earlier shown to generate ciNPCs from human, mouse, and somatic cells [49]) and postulated that this VCR cocktail with chemicals known to elicit the neurodifferentiation of neural progenitor cells can stimulate the transdifferentiation of human fibroblast cells into neural cells. Consistent with this, they found that Forskolin with VCR can partially induce a neural transdifferentiation activity in the human fibroblast. Besides these two molecules, they found that GO6983, SP600125, and Y-27632 can also efficiently promote neural transdifferentiation, which was validated by the presence of Tuj1^+^ cells with a neuronlike phenotype [53]. Although the cocktail of above seven small molecules demonstrated a distinct transdifferentiation efficiency, the survival and maturation of the produced neurons remained largely compromised. It led them to enroll extra neurotropic factors *viz.* NT3, BDNF, and GDNF along with CHIR99021, Forskolin, Dorsomorphin, which improved the method and produced fully functional and mature neurons. This method was further employed to produce functional neurons from familial AD patients. In another report, Zhang et al. identified a chemical cocktail that can efficiently transdifferentiate human astrocytes into neural cells [54]. Their screening involved a pool of 20 small molecules, compounds of which were earlier found to inhibit glial related signaling and to activate neural-related pathways/programs partly by modulating epigenetic programming. Extensive screening eventually led them to identify a cocktail of nine small molecules *viz.* SB431542, LDN193189, TTNPB, CHIR99021, Thiazovivin, DAPT, VPA, purmorphamine, and smoothened agonist that, in a stepwise manner, can efficiently transdifferentiate astrocytes into neurons. Of note, these transdifferentiated neurons sustained survival for about five months in vitro and constituted multiple functionally viable synaptic networks (Table 2 and Table 3).

Obtaining human cells and stem cells is a practical impediment. Given the non-invasive source of multiple types of cells, urine can be obtained from patients of any age. Urine cell-derived competent cells have emerged as a major tool for research given its therapeutic importance [55]. Xu et al. demonstrated direct transdifferentiation of human urine cells to neurons using a seven small molecule cocktail (CHIR99021, A8301, Y-27632, TTNPB, Forskolin, VPA, NaB) [56]. The transdifferentiated neurons exhibited a mature neuronlike phenotype and molecular signature as validated by the expression of neuronal markers. Further, Qin et al. used a combination of small molecules and protein factors and successfully performed transdifferentiation of human fibroblasts into neuronlike cells without passing through a neural stem/progenitor intermediate stage [35]. Although these reports showed efficient neuronal transdifferentiation from various cell types, the underlying molecular mechanism of these processes warrants further investigation.

#### 2.2.3. Other Chemical/Small Molecule-Mediated Transdifferentiation

Besides the cardiac and neuronal transdifferentiated studies, a few attempts have been made to obtain transdifferentiated pancreatic β-cell, endodermal, and liver progenitor cells [47,57,58,59]. However, this handful of reports represented preliminary results, and the authenticity of the transdifferentiated cells warranted further careful investigations. An early report suggested the in vivo transdifferentiation of pancreatic β-cell from other somatic cells with defined TFs [47]. Later, Fomina-Yadlin et al. reported that a compound *viz.* BRD7389 could potently transdifferentiate pancreatic α-cells to β-cells, as marked by similar phenotypic and gene expression signatures [57]. Katsuda et al. [59] revealed that the transdifferentiating activity of three small molecules, *viz.* A-83-01, Y-27632, and CHIR99021, could yield bipotent liver progenitor cells from mature hepatocytes. Of note, in vitro these liver progenitor cells can differentiate into biliary epithelial cells and active hepatocytes that can revive injured liver tissues in vivo. In another report, Wang et al. showed that a cocktail of defined chemicals, *viz.* Bay-K-8644, RG108, Bix01294, and SB431542, with tissue-specific mesenchymal feeders can facilitate transdifferentiation of human gastric epithelial cells to multipotent endodermal progenitor cells. These cells can further be systematically differentiated into hepatocytes, intestinal epithelial cells, and pancreatic endocrine cells [60].

## 3. Cellular Reprogramming in Disease Modeling

In 2006, the pioneering work of Yamanaka’s group demonstrated that forced expression of defined TFs *viz.* Oct4, Sox2, Klf4, c-Myc (OSKM; or Yamanaka factors) can instigate transcriptional reprogramming in murine somatic cells, converting them into induced pluripotent stem cells (iPSC) [61]. Replicating a similar strategy, human iPSCs were subsequently produced that revolutionized the field of regenerative medicine [62,63]. The human iPSCs were largely seen as a promising model for disease modeling and drug discovery, as these reflected the embryonic stem cells’ (ESCs) profile and had similar transcriptomic and chromatin signatures [63,64,65]. In a significant report, Cheng et al., working on murine fibroblasts and human urinary cells, for the first time successfully generated chemical-induced neural progenitor cells (ciNPCs) by treating cells with a three small molecules cocktail of valproic acid (VPA, i.e., propylpentanoic acid) —a HDAC inhibitor, CHIR99021—a GSK3 inhibitor—and Repsox—aTGFβ inhibitor [49]. They performed it under the physiological hypoxic (5% O_2_) conditions, having no expression of exogenous TFs/genes [49]. Alternatively, taking cocktails of LiCl, NaB, and SB431542 or Li_2_CO_3_, TSA, and Tranilast with HDAC, GSK3, and TGFβ inhibitors, they further showed similar effects for ciNPC induction and the state of multipotency as these cells became differentiated into different neuronal cells in vitro and in vivo. The cellular reprogramming capabilities of both sets of TFs and chemical models reflected their immense clinical utility in disease modeling [63,65,66,67] (Figure 2).

Cellular reprogramming refers to a group of approaches that allow researchers to halt or reverse the development of adult cells. The validation of cellular reprogramming in human cells has paved the way for a slew of new stem cell biology, disease modeling, drug development, and regenerative medicine applications [19]. The presence of pluripotent stem cells in a population that gives rise to all cells is one of the most defining elements of early mammalian development [68]. Due to a shortage of primary cells from the human central nervous system (CNS) and peripheral nervous system, human-induced pluripotent stem cells (hiPSCs) can also be studied for neurogenerative disease [69]. However, researchers have been able to conduct studies on the recapitulation of physiological and pathological pathways in patient-derived lines. This has resulted in more realistic disease modeling platforms [70]. These are widely utilized in drug discovery and safety investigations, for instance in the development of AD drugs with the goal of identifying chemicals that can inhibit or lower amyloid-beta levels [71] (Table 3 and Table 4).

**Figure 3:**
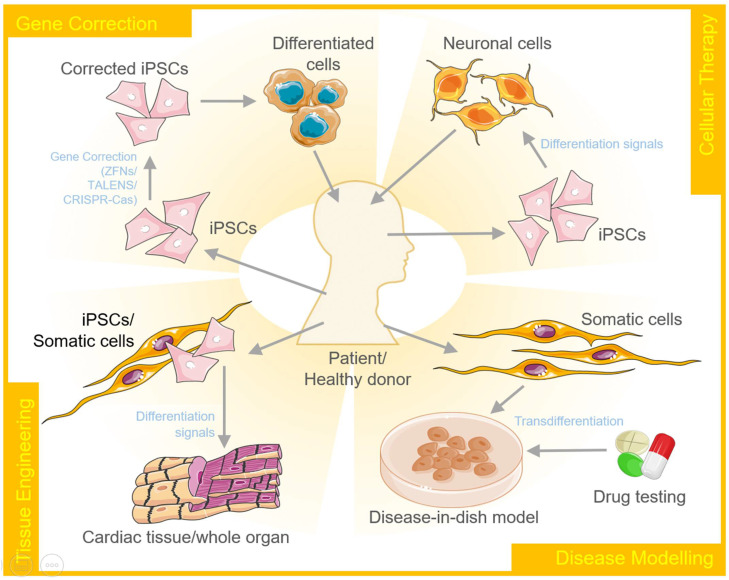
Schematic diagram is showing diverse therapeutic applications of transdifferentiation and cellular reprogramming in gene therapy/correction, cellular therapy, tissue engineering, and disease modeling.

Heart failure is another common illness that is linked to a high rate of morbidity and mortality. Coronary artery disease, genetic changes and mutations, viral infections, unfavorable immunological responses, and cardiac toxicity are the underlying causes [44]. This is due, in part, to roadblocks in translating research into treatment techniques. Drug repositioning using disease modeling systems based on hiPSCs could be a vital process that draws on prior toxicological and safety investigations to uncover new applications for existing medications [72]. The components of the reprogramming cocktail, as well as the administration method, have undergone significant changes throughout time to improve efficiency and offer a safer product. The current problems that stymie the clinical utility of cellular reprogramming and its applications in biomedical research [65], drug discovery, and predictive safety pharmacology are the explicit topics covered in this review (Figure 3).

### 3.1. Chemical/Small Molecule-Induced Cellular Reprogramming in Cardiomyocytes

In 2015, a study reported full chemical-induced fibroblast to cardiomyocytes reprogramming for the first time. In this report, Fu et al. aimed to perform chemical induction of iPSCs (CiPSCs) of mice fibroblast using a chemical cocktail *viz.* CRFVPT that included CHIR99021, RepSox, Forskolin, VPA, Parnate, TTNPB [73] (Table 3). In the course of this, they observed the spontaneous formation of contractile patterns in the cells, and their clusters that were phenotypically similar to cardiomyocytes. Such a phenotype was evident 6-8 days post-treatment with the CRFVPT cocktail, which was quicker than an earlier published work that exhibited the emergence of the CiPSCs phenotype in 20 days [73]. They systematically performed the transdifferentiation using a two-stage strategy wherein the CRFVPT cocktail was utilized to instigate the induction in first stage and cardiomyocytes-supporting medium, with CHIR99021, PD0325901, LIF, and insulin supplements, was used in the second stage. With this two-stage strategy, the CRFV regime was found to potently stimulate cardiac transdifferentiation to CiCMs that appeared to progress through a progenitor stage [73].

Cao et al., using the small molecules, performed the successful reprogramming of fibroblast cells into functional cardiomyocytes [74]. Using a fibroblast cell line derived from the human foreskin, they transduced αMHC-GFP reporter in the cells and screened a library of 89 compounds, taking as reference a baseline cocktail. They found that a 7C recipe including CHIR99021, A83-01, AS8351, BIX01294, SC1, Y27632, and OAC2 was enough to efficiently instigate cardiac reprogramming. Additionally, in an extended screening, they found that two small molecules, *viz.* SU16F and JNJ10198409, that are known to be the inhibitors of PDGF signaling, can enrich the reprogramming population. On evaluating its in vivo performance, they found that these chemically treated cells can be transplanted into injured/infarcted hearts and can efficiently convert into cardiomyocytelike cells in mouse models. In a recent report, Singh et al. recruited three small molecules, including sodium butyrate, ICG-001, and RA, along with GMT TFs and converted cardiomyocytes from primary rat heart fibroblasts [75]. The combinatorial approach was suggested to enhance the generation and induction of cardiomyocytes and offered an improvement of the cardiac regeneration practice for disease modeling (Table 3).

### 3.2. Embryonic and Induced Pluripotent Stem Cells for Disease Modeling

Embryonic development starts with a single cell, the zygote, and progresses through the steps of establishing diverse cell lineages, eventually assimilating cells into the embryonic structure. It facilitates a high level of inter/intra-cellular communication and developmental cues that generate tissues and organ patterns to eventually shape the entire body [77]. Smith and colleagues firstly evaluated the osteogenic differentiation of mouse embryonic stem cells (ESCs) in vitro after their collection from mice (mESCs) [78]. Xu and colleagues later reported the isolation of human ESCs (hESCs) [79]. However, the conditions necessary to sustain pluripotency and self-renewal of mESCs and hESCs in vitro are quite different. Therefore, adult somatic cell-derived induced pluripotent stem cells (iPSCs) are rapidly being examined as a less controversial patient-specific alternative to hESCs.

Importantly, iPSCs and ESCs have a high degree of similarity, providing new promise for the use of pluripotent stem cells for regenerative therapies with fewer ethical problems and potentially improved patient specificity [65] (Table 3). The development of innovative stem cell-based models to investigate the underlying processes of lineage differentiation and embryonic morphogenesis has been aided by the availability of embryo-derived stem cells that capture the lineage propensity [80].

Reprogramming the adult somatic cells into induced pluripotent stem cells (iPSCs) is another effective model that has a bright future as regenerative medicine. Therefore, disease models are critical for revealing the molecular basis of a variety of diseases, enabling the development of new treatments.

### 3.3. Cellular Reprogramming in Neuronal and Cardiac Disease Modeling

Neurons are the brain’s fundamental functional unit and are a diverse, dynamic, and important cell type in the study of cognitive function as well as the development of brain injury therapeutics [98]. Although the discovery of pluripotent transcription factors, including OSKM, were sufficient to efficiently transform mouse fibroblast cells into iPSCs [99], there are several drawbacks to existing iPSC technology, including the limited efficiency and a lengthy reprogramming process. Therefore, in molecular neurobiology, the direct reprogramming of somatic cells to distinct subtypes of induced neurons (iN) shows a lot of promise [100]. Certain transcription factor combinations are now known to directly create iN from a variety of cell types, which could be beneficial in the development of neurological illness models. This latest research is just the beginning of advancements that could allow us to use iN for disease modeling and medication in neurodegenerative diseases. The new generation of iN is crucial for understanding disease mechanisms and developing medications to treat neurodegenerative diseases (Table 4).

Human pluripotent stem cells (hPSCs) are also an extremely useful model system for studying the genetic basis of human cardiovascular disorders [67]. Unlike nonhuman animal models, human pluripotent stem cells (hPSCs) can be closely genetically matched to patients [101]. Hepatocytes, cardiomyocytes, and vascular endothelial and smooth muscle cells were all differentiated from hPSCs in large numbers and in a variety of cell types, which was crucial for determining the molecular underpinnings of the patient’s disease process [66]. Human embryonic stem cells (hESCs), first discovered in 1998, can be produced directly from human embryos. However, obtaining an hESC line often necessitates the destruction of an embryo that is unrelated to any live person [80]. As a result, induced pluripotent stem cells (iPSCs) are now the most important studies in which many types of human somatic cells have been effectively reprogrammed. One important goal related to patient-specific iPSCs will be to use or to create a cardiac-based model system that incorporates all the genetic loci involved in the pharmaceutical response [101] (Table 3). Clinically significant mutations can be extracted from the cells of patients suffering from a specific genetic disorder. One such study was performed considering the phenotype of iPSC-derived cardiomyocytes (iPSC-CMs) obtained from patients. These patients were more susceptible to catecholamine-induced tachyarrhythmia that was reduced by beta-blockade therapy [102]. These findings show that iPSCs may reliably recreate aberrant cellular phenotypes and behaviors in vitro, offering vital mechanistic insights into the disease process. It will be extremely valuable not only for disease modeling research (Table 1) but also for prospective clinical applications and high-content, large-scale drug screening methods (Table 3 and Table 4).

### 3.4. Present Status, Developments, and Emerging Reprogramming Trends

Pluripotent stem cells (PSCs), which include embryonic stem cells (ESCs) and induced pluripotent stem cells (iPSCs), have a limitless ability to self-renew and proliferate. This feature allows them to generate a therapeutically relevant number of cells for regenerative therapy [24]. This would help the researchers to better understand the mechanisms driving a variety of human genetic, malignant, and nonmalignant disorders. Genome editing techniques have also been utilized to fix disease-specific iPSC mutations, resulting in gene-corrected iPSCs that can be employed for autologous cell-based treatment [103]. The number and kind of cells, their efficiency, footprint, and long-term translational goal influences all its reprogramming approaches. However, fibroblasts and peripheral blood mononuclear cells remain the gold standard, despite the usage of diverse cell types. When compared to iPSCs produced from other parental tissues, blood cells were less likely to develop aberrant DNA methylation, and these cells exhibited stronger hematopoietic differentiation ability [104,105]. Therefore, the generation of patient-specific iPSCs provides a safer alternative for clinical applications.

Transgenic animal models have traditionally been used to investigate disease pathogenesis but due to inherent variations across species, many of these models do not completely recreate illness characteristics [106]. As a result, most research has depended on the investigation of disease pathology using peripheral blood cells, which have a short lifespan in culture [104]. Genetic alterations, which are key tools for studying candidate gene function, are also hampered by the lack of a strategy to maintain and amplify the primary cells. The introduction of iPSC technology has revolutionized how we investigate reprogramming models and their derivatives for illuminating pathogenic events during disease start and progression. It would otherwise go undetected in primary cells [107]. Furthermore, in neuronal disorders, essential steps in generating and converting stem cell therapies from the bench to patients include identification of the proper stem cell type and understanding the mechanism of support [88]. There is substantial evidence that stem cell therapy can improve neurogenesis in patients with neurological diseases [108]. Cell line-based chemical screening and animal testing have been used to develop a huge number of medications currently on the market [109]. However, numerous medications failed to reach the market due to unanticipated side effects in late-stage trials, primarily cardiotoxicity and hepatotoxicity. High-throughput screening assays against a library of hundreds of thousands of chemicals are possible because of a wide panel of disease-specific iPSCs and their derivatives [110]. This method may make it easier to design new treatments. Disease modeling is a crucial technique for elucidating the molecular basis of a variety of diseases and enabling the development of new targeted therapeutics. The concept of “disease in a dish” using ESCs/iPSCs has opened up new avenues for the experimentally derived understanding of disease mechanisms, paving the way for new targeted therapy alternatives [111]. Despite advancements in iPSC technology, the absence of efficient induction techniques continues to limit the creation of these cells [24]. Cost/resource needs, as well as the tendency of iPSCs to return to the genetic makeup of the original somatic cell type over time, are all ongoing concerns [112]. The present cellular reprogramming methods highlight the need for better stem cell production procedures. As a result, one of the ways that researchers developed was DeepNEU, a revolutionary unsupervised deep-machine learning framework for simulating iPSCs and enabling effective cellular reprogramming [113]. It was confirmed by creating computer simulations of three iPSC models that had previously been produced experimentally and published in peer-reviewed journals. The use of this computer technology to generate disease-specific artificially induced pluripotent stem cells (aiPSCs) has the potential to improve: (1) disease modeling; (2) rapid prototyping of wet-lab experiments; (3) grant application writing; and (4) specific biomarker identification, all at a low cost. This potential new technique is still being developed and validated, with the present focus on modeling rare genetic illnesses [65,113]. This shows that in the near future transdifferentiation and reprogramming therapies are expected to be successfully put into clinical practice.

## 4. Therapeutic Applications of Transdifferentiation and Cellular Reprogramming

### 4.1. Disease Modeling and Testing Therapeutics

A disease model represents the abnormal state of cells that occur in a specific disease. Therefore, it allows researchers to investigate and understand the intricate mechanisms that lead to the onset and further progression of the disease. These models can further be explored for developing and testing therapeutics. Cellular reprogramming of stem cells to create *disease-in-a-dish* models has gained a lot of attention over the past few years. These disease models are capable of self-renewal and also differentiate into desired cellular types to capture the disease pathogenesis [114] (Figure 3).

It was the use of ESCs derived from affected embryos that gave insights into the early developmental events of Fragile X Syndrome (FXS), the most common genetic cause of autism. It was found for the first time that FMR1 silencing of the mutated gene in an X-linked dominant manner happens only after differentiation and not in the embryonic stage [115]. Using iPSCs, one of the earliest disease models developed was to study spinal muscular atrophy. The motor neurons produced by diseased iPSCs carried the histological markers of the disease and degenerated at a rate faster than the wildtype control neurons [116]. A disease model for schizophrenia established using iPSCs derived from patients with a 4 bp deletion in the DISC1 locus has also been reported. This is one of the successful examples where the episomal vector approach was used for the generation of adult patient-derived iPSCs without the risk of insertional mutagenesis [117]. Along similar lines, disease models for many other neurological disorders have been developed to date, including Rett syndrome [118], Down syndrome [119], Parkinson’s disease [120], and Alzheimer’s disease [121]. This iPSC technology has also been used to model various cardiac diseases including long QT syndrome, Leopard syndrome, Brugada syndrome, catecholaminergic polymorphic ventricular tachycardia, arrhythmogenic right ventricular cardiomyopathy/dysplasia, dilated cardiomyopathy, left ventricular non-compaction, hypertrophic cardiomyopathy, Andersen–Tawil syndrome, Timothy syndrome, Friedreich’s ataxia, Barth syndrome, fatty acid oxidation disorders, and Pompe diseases. All these models have been discussed in detail in another report [122].

Organ-on-chip technology is another area showing progression at an accelerated rate. A group of researchers has generated a blood–brain barrier chip by integrating iPSCs and organ-on-chip technologies. These chips can serve good as a disease model for CNS drug penetrability predictions [123]. Another example is of a four-organ-chip consisting of interconnected miniaturized human intestine, liver, brain, and kidney equivalents created using pre-differentiated iPSCs. Three out of four models (intestine, liver, and neuronal) were shown to maintain defined marker expression under specific conditions for over 14 days; however, the renal model could not succeed as expected [124]. Nonetheless, these efforts expedite new avenues for modeling microphysiological systems. These systems can effectively be used as autologous coculture crosstalk assays, understanding the disease mechanisms, and for further using the platform for drug testing.

Along with providing mechanistic insights, these disease models have also been successfully employed to screen therapeutics. Many drug candidates that appear to be effective and safe in preclinical cellular and animal models often fail when tested in human beings. This can be attributed to the non-reliability of the preclinical models in practice. These models fail to recapitulate human physiology to its best. The popular cellular models for testing drug candidates include Chinese hamster ovary (CHO) cells and human embryonic kidney (HEK) cells. However, these cells do not reflect some of the important characteristics of human cardiomyocytes and also lack the expression of ion channels other than hERG. This often leads to incorrect assessments of drug effects. For instance, the action of Alfuzosin is mediated through sodium channels rather than hERG and causes QT prolongation. So, it appears to be non-toxic when tested in hERG-overexpressing cell lines [125]; however, testing in iPSC-CMs, it was identified as toxic [126]. This example thus clearly demonstrates the importance of correct screening models. Next, Long QT syndromes (LTQS) are caused by mutations in KCNQ1 or KCNH2. These genes encode voltage-gated potassium channels. The disease model for this syndrome, generated from iPSC-derived cardiomyocytes, showed significantly longer action potentials in comparison to the control cells. The dominant-negative trafficking effect of KCNQ1 and the resulting reduction in rectifier current was also studied through this model. Testing of known tests for this syndrome was then followed by the identification of β-adrenergic receptor inhibitors, nifedipine, and pinacidil as novel therapeutic candidates [127,128]. iPSC-derived disease models can also be effectively used for high-throughput screening assays. A study reported the use of neural stem cells differentiated from FXS patient-derived iPSCs in a 1536-well plate format high-throughput screening. Out of 5000 tested compounds, the authors were able to identify six compounds that modestly increased FMR1 gene expression in the patient-derived cells. Though the results did not provide any clue on their clinical application the study still proves the principle in question [129]. Next, iPSC-CMs have been reported to be employed for the screening of 131 different drugs at 6 different concentrations for the cardiotoxicity test [130]. Another similar study evaluated 51 previously characterized compounds in iPSC-CMs to study their effects on cardiomyocyte contraction [131]. The US FDA has also used iPSC-CMs for the evaluation of drug-induced arrhythmic risk [132]. Further, a liposomal formulation of doxorubicin was also tested in a similar model. Doxorubicin in this new formulation was unable to penetrate cardiomyocytes, thereby approving its entry into phase I clinical trials [133].

### 4.2. Regenerative Medicine

The concept of regenerative medicine involves the switching of stem cells or dedifferentiating somatic cells into stem cell-like multipotent cells. These cells can proliferate and then re-differentiate into the desired lineage to repopulate the damaged or degenerated tissue with functional cells. The reprogramming of the cells can be conducted in vitro, in vivo, or ex vivo to regain their regenerative properties. The use of a single transcription factor, such as FOXN1, has been shown to regenerate the thymus in aged mice. Though a lot of efforts are being made to explore and understand mammalian stem cell biology, the knowledge regarding the regenerative capacity of the mammalian system is still limited. However, it is known that the cellular environment, including the modulators present in the extracellular matrix, cytokines, and growth factors, plays a crucial role in this process [134] (Figure 3).

There are plenty of examples that demonstrate the immense potential of regenerative medicine. The first clinical trials using stem cell-derived products for treating neurological diseases in human were initiated by Geron in 2009. The system used hESCs-derived oligodendrocytes to remyelinate denuded axons in patients with thoracic level spinal injury. However, the study faced preclosure due to financial concerns [135]. Since then, a lot of reports have been published that explore the utility of ESCs or iPSCs. In a more recent study, intervertebral disc-derived iPSCs differentiated into neural precursor cells were transplanted into mice to investigate the effect on spinal injury. The precursor cells were observed to differentiate into early and matured neurons, significantly improving the hindlimb dysfunction in the injured mice [136]. Making use of cell therapy for Parkinson’s disease requires the establishment of methods to produce human iPSCs or hESCs-derived midbrain-type dopaminergic neurons. Sundberg et al. developed a method for the same that resulted in the restoration of motor function in treated rats [137]. On similar grounds, iPSCs generated from nonobese diabetic mouse embryonic fibroblasts and pancreas-derived epithelial cells were reprogrammed to differentiate into functional pancreatic β-cells. These cells were further seen to produce insulin when transplanted into diabetic mice, thereby normalizing hyperglycemic conditions and paving way for treating diabetes [138]. The potential utility and feasibility of ESC or iPSC-derived cardiomyocyte transplantation therapy for myocardial regeneration have also been discussed in many reports. It has been shown that embryonic-stem-cell-derived cardiomyocytes, when transplanted into a guinea-pig model with an injured heart, protected it against arrhythmias. The grafted muscles were also able to contract synchronously with the host muscle, improving the mechanical function of the injured heart and reducing the risk of ventricular tachycardia [139]. Though many other successful reports have come to light, we are discussing only a few to emphasize the clinical applications of this approach.

An alternative to the natural regenerative potential of mammalian stem cells is to induce transdifferentiation in somatic cells. Differentiated cells, such as neurons derived from iPSCs, have been observed to represent an embryolike stage. The epigenetic changes that a cell undergoes as it ages or becomes diseased are therefore not reflected by the matured cells. This results in the importance of the transdifferentiation process, whereby the phenotype of one somatic cell type can be converted into another without an intermediate progenitor stage [73]. For instance, Ieda and his colleagues used a combination of Gata4, Mef2c, and Tbx5 developmental transcription factors to transdifferentiate postnatal cardiac or dermal fibroblasts into cardiomyocytelike cells. The gene expression profile and function of the differentiated cells was also found to be similar to the adult cardiomyocytes [140]. Similar studies enhancing the in vivo efficiency of cardiac cell reprogramming [141] and the use of small molecules for the same have also been reported [74].

### 4.3. Tissue Engineering

In simple words, tissue engineering refers to the in vitro production of tissues or organs for clinical applications using stem cell-derived differentiated cells. This technology is already in use, mainly for skin replacement and cartilage repair [142]. iPSCs and ESCs serve as an excellent source to produce desired cell types using cellular reprogramming or transdifferentiation (Figure 3).

iPSC-derived multipotent neural crest stem cells (NCSCs) supported by scaffold fabrication have previously been used to engineer nerve conduits for regenerating the sciatic nerve [143]. The rolling advancements in studies related to biomaterials, scaffold fabrication, 2D- and 3D-bioprinting have further accelerated development in the field of tissue engineering. Following this are some more examples to support this statement. Transdifferentiated endothelial cells capable of angiogenesis have been successfully utilized for re-endothelialization in tissue-engineered vessels [144]. Next, progress was made by utilizing the smooth muscle cells to generate functional endothelial cells [145]. Attempts have also been made at reprogramming hepatocytes for regenerating livers in mice [146]. This method was improved in a parallel study, and it resulted in the generation of transdifferentiated hepatocytes capable of synthesizing and excreting bile acid [147]. These developments can pave the way for treating cholestatic diseases as well. A recent study has further evolved the field by showing the generation and self-organization of kidneylike structures using hPSCs-derived renal cells. These organoids also contained functional glomeruli [148].

### 4.4. Gene Editing

Gene editing, in combination with stem cell technology, has the potential to revolutionize the field of medicine, especially for the corrective therapy of monogenic diseases such as sickle cell anemia. In the most simplistic representation, it works by generating patient-specific iPSCs, correcting the genetic defect, ex vivo/in vivo differentiation of the modified cells, followed by the transplantation of the corrected cells/tissue into the patient. The discovery of gene-editing tools, such as zinc finger nucleases, TALENS, and CRISPR/Cas9 has relatively simplified the process of gene editing, making the dream come true (Figure 3).

One of the encouraging examples here is the replacement of CAG expansion with normal repeats in the huntingtin gene in iPSCs derived from fibroblast cells of Huntington patients. The correction was sustained by the differentiation of DARPP-32-positive neurons as well under both in vitro and in vivo conditions [149]. A similar approach has also been used for correcting mutations in iPSCs derived from patients suffering from A1AT deficiency [150], sickle cell anemia [151], tauopathy [152], spinal muscular atrophy [153], and β-thalassemia [154].

Along with correcting the diseased phenotype, this combination of technologies can also be used to create and study genotypic alterations rarely found in the population. Thus, gene editing can be used to introduce disease-causing mutations into iPSCs or human ESCs from healthy donors. A group of researchers has used TALENs to mutate 15 different genes known to cause different disorders, including dyslipidemia, insulin resistance, hypoglycemia, lipodystrophy, motor neuron death, and hepatitis C virus infection [155].

### 4.5. Personalized Medicine

With advancements in next-generation sequencing technologies and cost-cutting, personalized medicines will soon become a routine procedure. With an increase in our understanding of stem cell biology and the evolution of genome editing tools, it is anticipated that the global vision of being able to treat almost any disease will soon become a reality. The above sections discussed many examples where patient-derived iPSCs have already been reprogrammed and differentiated into varied cell types depending upon the experimental/clinical objective (Figure 3).

iPSCs offer an efficient system for testing the effect of drugs in patients harboring different disease-associated mutations. The expected response of cardiovascular drugs such as warfarin, clopidogrel, and statins has been shown to be affected by the genotype of the patient. Therefore, choosing the right drug at the right dose for the right patient at the right time is one of the most important goals of precision medicine. As a supporting example, iPSC-CMs generated from patients with LQTS or hypertrophic cardiomyopathy were found to be more vulnerable to the arrhythmogenic effects of cisapride [156]. In another study, two potential I_Ks_ blockers increased the toxicity in iPSC-CMs from LQTS2 patients when compared to the control cells [157]. In yet another study, a patient with LQTS and frequent ventricular arrhythmias was found to have mutations in KCNH2 andSCN5A. The iPSC-CMs derived from this patient led to the finding that disease phenotype here was due to defects in the sodium channels and not the potassium channels. The patient was therefore administered mexiletine alone, a sodium channel blocker. The patient’s arrhythmias were controlled without the need for a secondary sodium channel blocker, flecainide [158].

Not only this, as described above, it has now become possible to create patient-specific iPSCs-derived disease models to understand the intricate disease mechanisms. Additionally, it is now feasible to monitor the effectiveness of specific drugs or their side-effects for informed therapeutic decisions. iPSC-CMs from 13 individuals were used to investigate the cardiotoxicity of 21 FDA approved tyrosine kinase inhibitors (used for treating various types of cancer) and generate a cardiac safety index [159]. Patients with monogenic genetic disorders can also benefit from gene-editing technologies. Another potential personalized application is to model a platform and assess the individual’s risk from specific environmental hazards. In one study, iPSC-CMs have also been used as an in vitro model for coxsackievirus B3–induced myocarditis [160].

Considering the clinical potential of iPSCs-based studies in clinical trials, in Table 5 we comprehensively listed some of the promising studies (including disease modelling, drug screening, and therapy-based studies) already in clinical trials. More details related to the experimental design and expected outcomes of each of the listed study can be accessed via https://www.clinicaltrials.gov/ (accessed on 25 July 2021) using the associated NCT number.

## 5. Conclusions

Cell transdifferentiation and induced pluripotent stem cells have opened countless avenues with advancements in technologies that make it possible to reprogram these to differentiate into desired cell types. This field was further revolutionized by the development of methods that allow the direct transdifferentiation of somatic cells into a different cell lineage without the intermediate pluripotent state. This review discussed the various possible applications of these methods. It will soon be possible to study and understand any disease or disorder frequently existing or rarely found in a population. A wide variety of disease models have been developed to date with an inclination towards neurological, cardiac, hepatic, and cancer models. These transdifferentiated and induced models are also being applied for testing drugs and their efficient delivery into cells. A combination of the strength of iPSCs or hESCs with genome editing tools is also helping the scientific community to regulate genetic defects. Moreover, this cell fate differentiation technology is yet another step up in realizing the dream of personalized medicine for humankind.

## Figures and Tables

**Table 1:** Transcriptional factor(s)(TFs)-induced transdifferentiation, mechanism of action, and outcomes in the CNS/brain and cardiac model systems.

	Disease Model	Species/Tissue	Source (Cell Type)	Transdifferentiated Cell (Converted)	Transdifferentiation Factors/TFs	Delivery/Vehicle	Efficiency	Results/Physiological Outcome	References
CNS/Brain	-	Mouse brain	Astrocyte, Fibroblast	Induced neuron (iN)	*Myt1l, Ascl1, Brn2a*	Transduced in vitro/Doxycycline in drinking water/Lentiviral	0.4% to 5.9%	iNs in tissue	[27]
			CPN (Colossal projection neuron)	Corticofugal projection neuron (CFuPN)	*Fezf2*	Electroporation (in utero)/Plasmid DNA	75.6% of Fezf2-expressing CPN (+ve for CFuPN marker)	Morphological change, gene and protein expression (until P3). Axon connectivity change (until E17.5)	[33]
			L4 neuron	L5B neuron	*Fezf2*	Electroporation (in utero)/Plasmid DNA	50% for Fezf2+ L4 and L5B neurons	Morphological change, gene and protein expression (until P1)	[34]
			Astrocyte	Induced adult neuroblast (iANB)	*Sox2*	Stereotactic (brain)/Lentiviral	41% of YFP+ cells expressed NG2; 23.2% of GFP+ cells were +ve for DCX	Transdifferentiated iANBs in tissue. Transdifferentiated mature neurons (+BNTP/Noggin or VPA)	[28,29]
	Stab injury in AD	Mouse brain, Human cortical astrocytes	Astrocyte NG2 cell	Induced neuron (iN)	*NeuroD1*	Stereotactic (brain)/Retroviral	90%	Transdifferentiated iN in tissue. Functional or behavioural data not acquired	[32]
	Spinal cord injury	Mouse Spinal cord	Astrocyte	Induced adult neuroblast (iANB)	*Sox2*	Stereotactic (spinal cord)/Lentiviral	3–6% were reprogrammed by SOX2	Transdifferentiated iANBs in tissue. Mature neurons (+VPA) synapses with resident neurons. Functional data not acquired	[30]
Cardiac	Freeze–thaw injury	Rat	Cardiac fibroblasts	Skeletal myofibers	MyoD	Intramyocardial/Adenovirus	2–14%	Produced myofibers (immature) in tissue	[8]
	Myocardial infarction	Mouse	Cardiac fibroblast	Cardiomyocytes	GMT (Gata4, Mef2c, Tbx5)	Intramyocardial/Retrovirus	10–15%	Reduced infarct size. Significant decrease in cardiac dysfunction	[37]
			Cardiac fibroblast	Cardiomyocytes	GMT (Gata4, Mef2c, Tbx5)	Intramyocardial/Retrovirus	3–7%	Cardiomyocytelike cells in fibrotic area. No phsiological functional results	[38]
			Cardiac fibroblast	Cardiomyocytes	GHMT (Gata4, Hand2, Mef2c, Tbx5)	Intramyocardial/Retrovirus	~7%	Reduced infarct size. Significant decrease in cardiac dysfunction	[39]
			Cardiac fibroblast	Cardiomyocytes	microRNAs 1, 133, 208 & 499	Intramyocardial/Lentivirus	12–25%	Fibroblast transdifferentiation into cardiomyocyte in the infarct spot/area. Moderate decrease in cardiac dysfunction	[40,41]
	Complete heart blockage	Pig	Ventricular cardiomyocytes	Pacemaker cell-induced sinoatrial node cells	Tbx18	Percutaneous, to heart ventricule/Adenovirus	24.5%	Constituting a biological pacemaker. Improvement of bradycardia	[42]
			Ventricular cardiomyocytes	Pacemaker cell-induced sinoatrial node cell	Tbx18	Intramyocardial/Adenovirus	9.2%	Constituting a biological pacemaker. Improvement of bradycardia	[43]

**Table 2:** List of cardiovascular diseases modeled with human iPSCs or ESCs.

Disease Model for Cardiac Dysfunctions	Impacted/Mutated Genes
Left ventricular noncompaction	TBX20, GATA4
Familial hypercholesterolemia	LDLR, PCSK9
Timothy syndrome	CACNA1C
Dilated cardiomyopathy	TTN, TNNT2, LMNA, DES
Duchenne muscular dystrophy	DMD
Arrhythmogenic right ventricular dysplasia	PKP2
Long-QT syndrome type 1	KCNQ1
Jervell and Lange-Nielsen syndrome	KCNQ1
Catecholaminergic polymorphic ventricular tachycardia type 1	RYR2
Catecholaminergic polymorphic ventricular tachycardia type 2	CASQ2
Brugada syndrome	SCN5A
Calcific aortic valve	NOTCH1
Williams–Beuren syndrome	ELN
Familial pulmonary hypertension	BMPR2
Barth syndrome	TAZ
Hypertrophic cardiomyopathy	MYH7
Maturity-onset diabetes of the young type 2	GCK
Insulin resistance	AKT2
Familial hypobetalipoproteinemia	PCSK9
Long-QT syndrome type 2	KCNH2
Long-QT syndrome type 3	SCN5A
Tangier disease	ABCA1
Dyslipidemia	SORT1
Hypoinsulinemic hypoglycemia and hemihypertrophy	AKT2

**Table 3:** Chemicals/small molecules-induced cellular reprogramming and their molecular activity/function(s) in neuronal and cardiac model systems.

Chemicals/Small Molecules	Molecular Activity/Induced Mechanism(s)	Cellular Reprogramming Function(s)	References
RepSox (E-616452)	TGF-βRI (ALK5) inhibitor	CiNPC, CiN, CiCM	[49,53,73]
TTNPB	RAR ligand	CiCM, CiN	[54,73]
Forskolin	Adenylyl cyclase activator	CiN, CiCM	[52,53,73]
CHIR99021	GSK3 inhibitor	CiNPC, CiNSLCe, CiNf, CiCM	[52,53,54,73,74,76]
VPA	HDAC inhibitor	CiPSCa, CiNPCb, CiNc, CiCMd	[49,53,54,73]
LiCl and Li2CO3	GSK3 inhibitor	CiNPC	[49]
SB431542	TGF-βRI inhibitor	CiPSC, CiNPC, CiN, hiEndoPC	[49,54]
NaB	HDAC inhibitor	CiNPC	[49]
Tranilast	Inhibit TGF-β1 secretion	CiNPC	[49]
TSA (Trichostatin A)	HDAC inhibitor	CiNPC	[49]
RG108	DNA methyltransferase inhibitor	CiNSLC	[50]
A-83-01	TGF-βRI (ALK4/5/7) inhibitor	CiNSLC, CiCM	[50,74]
Hh-Ag 1.5	Smoothened agonist	CiNSLC	[50]
SMER28	Autophagy modulator	CiNSLC	[50]
Retinoic acid	RAR ligand	CiNSLC	[50]
LDN193189	BMP type I receptor (ALK2/3) inhibitor	CiNSLC	[50]
GO6983	PKC inhibitor	CiN	[53]
ISX9	neurogenesis inducer	CiN	[52]
Dorsomorphin	AMPK and BMP I receptor inhibitor	CiN	[53]
I-BET151	BET inhibitor	CiN	[52]
SP600125	JNK inhibitor	CiN	[53]
SAG	Smoothened agonist	CiN	[54]
Y-27632	ROCK inhibitor	CiN, CiCM	[53,74]
Purmorphamine	Smoothened agonist	CiN	[54]
DAPT	Gamma-secretase inhibitor	CiN	[54]
SC1	ERK1 and RasGAP inhibitor	CiCM	[74]
Thiazovivin	ROCK inhibitor	CiN	[54]
OAC2	Epigenetic modulation	CiCM	[74]
AS8351	Epigenetic modulator	CiCM	[74]
SU16F	PDGFR-β inhibitor	CiCM	[74]
JNJ10198409	PDGFR-α and PDGFR-β inhibitor	CiCM	[74]
Bix01294	Histone methyl transferase inhibitor	CiCM	[74]

CiN: chemical-induced neuron; CiNPC: chemical-induced neuroprogenitor cell; CiCM: chemical-induced cardiomyocyte; CiNSLC: chemical-induced neural stem cell-like cell.

**Table 4:** TFs-induced cellular reprogramming and functional outcomes in neuronal and cardiac model systems.

	Reprogramming Factors (TFs)	Species/Model/Cell Type	Obtained Cell Types	Efficiency	Results/Functional Outcome	References
Neuronal	*Brn2, Myt1l, Zic1, Olig2, and Ascl1*	Mouse embryonic and postnatal fibroblast cells	iN (mostly GABAergic and glutamatergic neurons)	∼50%	Synaptic maturation, functional electrophysiology	[76]
	*Ascl1, Brn2 and Myt1l*		iN (mostly excitatory neurons)	19.50%	Synaptic maturation, functional electrophysiology	[76,81]
	*Forskolin, ISX9, CHIR99021 and SB431542*	Mouse fibroblast cells	iN	>90%	Functional electrophysiology	[52]
	*Ascl1, Brn2, Myt1l*	Mouse hepatocytes	iN	>90%	Functional electrophysiology	[82]
	*Mash1, Nurr1 and Lmx1a*	Mouse and human cells/fibroblast cells	iN (mostly dopaminergic neurons)	High	-	[83]
	*Ascl1, Brn2 and Myt1l*		neurons	20%	Functional	[27]
	*Sox2 and Mash1*	Pericyte-derived cells of the adult human cerebral cortex	GABAergic neurons	∼50%	Obtained iN acquire the ability of action potential firing, synaptic targets for neurons	[84]
	*LDN193189, SB431542, TTNPB, Tzv, CHIR99021, VPA, DAPT, SAG, Purmo*	Human astrocytes	Functional neurons (mainly glutamatergic neurons)	>90%	Functional	[54]
	*ASCL1, NGN2, SOX2, NURR1 and PITX3*	Human fibroblast cells	iN (mostly dopaminergic neurons)	∼80%	Functional electrophysiology	[85]
	*NeuroD1, Ascl1, Brn2, and Mytl1*		iN	∼60%	Functional neurons	[81]
	*Ascl1, Lmx1a, FoxA2, and FEV*		serotonergic (i5HT) neurons	∼25%	Showed spontaneous electrophysiological activity, Active synaptic transmission observed	[86]
Cardiac	*GATA4, MEF2C, TBX5, HAND2*	Mouse	iCMs from MEFs	~70–80%	Spontaneous beating, Ca^2+^ transients	[87]
	*GATA4, MYOD-MEF2C, TBX5, HAND2*		iCMs from embryonic head fibroblasts	10-20%	Spontaneous beating, Ca^2+^ transients	[88]
	*GATA4, MEF2C, TBX5, HAND2, NKX2.5, SB431542*		iCMs from MEFs	17%	Spontaneous beating, Ca^2+^ transients	[89]
	*MEF2C, GATA4, TBX5*		iCMs from CFs	~10%	Action potentials, spontaneous beating, Ca^2+^ transients	[38]
	*GATA4, MEF2C, TBX5, HAND2, miR-1, miR-133, A83-01, Y-27632*		iCMs from MEFs	60%	Action potentials, spontaneous beating, Ca^2+^ transients	[90]
	*GATA4, MEF2C, TBX5, (HAND2), Bmi1 shRNA*		iCMs from CFs	22%	Spontaneous beating, Ca^2+^ transients	[91]
	*GATA4, MEF2C, TBX5, SB431542, XAV939*		iCMs from CFs	~30%	Spontaneous beating, Ca^2+^ transients	[92]
	*GATA4, MEF2C, TBX5, HAND2, DAPT*		iCMs from MEFs	~38%	Ca^2+^ transients, spontaneous beating	[93]
	*GATA4, MEF2C, TBX5, MESP1, MYOCD*	Human	iCMs from HCFs	5.90%	Ca^2+^ transients, action potentials	[94]
	*GATA4, MEF2C, TBX5, ESRGG, MESP1, MYOCD, ZFPM2*		iCMs from hESC-derived fibroblasts	13%	Ca^2+^ transients, action potentials	[95]
	*GATA4, MEF2C, TBX5 (+ MESP1, MYOCD) with miR-133*		iCMs from HCFs	27.80%	Ca^2+^ transients	[96]
	*GATA4, MEF2C, TBX5, (HAND2, MYOCD or miR-590)*	Human, rat, porcine	iCMs from adult HCFs	~40%	No spontaneous beating in human iCMs	[97]

**Table 5:** Examples of iPSCs-based studies in clinical trials (Row 1 to 20—Disease modelling; Row 21 to 23—Drug screening; Row 24 to 31—Therapy based studies). More details related to the experimental design and expected outcomes of each of the listed study can be accessed from https://www.clinicaltrials.gov/ (accessed on 25 July 2021) using the associated NCT number.

	S. No.	NCT Number	Title	Disease Condition	Phase	Location of Study
Disease modelling	1	NCT02564484	iPSC Neurons From Adult Survivors of Childhood Cancer Who Have Persistent Vincristine-Induced Neuropathy	Leukemia|Lymphoma	Unknown	St. Jude Children’s Research Hospital, Memphis, Tennessee, United States
	2	NCT01860898	A Phase I Study of iPS Cell Generation From Patients With COPD	Thoracic Diseases|Respiratory Tract Diseases|Cancer of Lung|Cancer of the Lung|Lung Cancer|Lung Diseases, Obstructive|COPD|Pulmonary Emphysema|Neoplasms, Lung|Neoplasms, Pulmonary|Pulmonary Cancer|Pulmonary Neoplasms|Carcinoma, Non-Small-Cell Lung|Carcinoma, Small Cell	Not Applicable	Mayo Clinic in Rochester, Rochester, Minnesota, United States
	3	NCT02980302	Development of the Tool “ iPSC “ for the Functional Study of Mutations Responsible for Mental Retardation	Intellectual Deficiency|Asymptomatic Carrier of the Mutation of the Gene MYT1L|Healthy Volunteers	Not Applicable	UniversityHospitalGrenoble, La Tronche, France
	4	NCT02193724	Feasibility of Generating Pluripotent Stem Cells From Patients With Familial Retinoblastoma	Retinoblastoma	Unknown	St. Jude Children’s Research Hospital, Memphis, Tennessee, United States
	5	NCT02162953	Stem Cell Models of Best Disease and Other Retinal Degenerative Diseases	Retinal Disease|Bestrophinopathy|Best Vitelliform Macular Dystrophy|Adult Onset Vitelliform Macular Dystrophy|Autosomal Dominant Vitreoretinalchoroidopathy	Unknown	Mayo Clinic, Rochester, Minnesota, United States
	6	NCT03883750	Induced Pluripotent Stem Cells for Niemann Pick Disease	Niemann–Pick Diseases	Unknown	Childrens Hospital and Institute of Child Health, Ferozepur Road, Lahore, Pakistan
	7	NCT03867526	Establishment of Human Cellular Disease Models for Wilson Disease	Wilson Disease	Unknown	Childrens Hospital and Institute of Child Health, Ferozepur Road, Lahore, Pakistan
	8	NCT03754088	In vitro Model of the Cystic Fibrosis Bronchial Epithelium Via iPS Technology	Cystic Fibrosis	Unknown	HÃ’pital Arnaud de Villeneuve—CHU de Montpellier, Montpellier, France
	9	NCT01534624	Stem Cell Study of Genetics and Drug Addiction	Induced Pluripotent Stem Cells	Unknown	National Institute on Drug Abuse, Baltimore, Maryland, United States
	10	NCT01865981	Investigating Hereditary Cardiac Disease by Reprogramming Skin Cells to Heart Muscle	Eletrophysiology of iPS-derived Cardiomyocytes	Unknown	University of Dundee, Dundee, Angus, United Kingdom
	11	NCT03872713	Establishment of Human Cellular Disease Models for Morquio Disease	Morquio Disease	Unknown	Childrens Hospital and Institute of Child Health, Ferozepur Road, Lahore, Pakistan
	12	NCT01639391	Creation of a Bank of Fibroblast From Patients With Amyotrophic Lateral Sclerosis: Pilot Study	Amyotrophic Lateral Sclerosis	Not Applicable	Centre rÃ©fÃ©rent maladies rares SLA, Paris, France
	13	NCT03898817	Pathology of Helicases and Premature Aging: Study by Derivation of hiPS	Age Problem	Unknown	University Hospital Montpellier, Montpellier, France
	14	NCT01517425	Evaluating Cardiovascular Phenotypes Using Induced Pluripotent Stem Cells	Coronary Artery Disease	Unknown	Scripps Translational Science Institute, La Jolla, California, United States
	15	NCT02413450	Derivation of Human Induced Pluripotent Stem (iPS) Cells to Heritable Cardiac Arrhythmias	Inherited Cardiac Arrythmias|Long QT Syndrome (LQTS)|Brugada Syndrome (BrS)|Catecholaminergic Polymorphic Ventricular Tachycardia (CPVT)|Early Repolarization Syndrome (ERS)|Arrhythmogenic Cardiomyopathy (AC, ARVD/C)|Hypertrophic Cardiomyopathy (HCM)|Dilated Cardiomyopathy (DCM)|Muscular Dystrophies (Duchenne, Becker, Myotonic Dystrophy)|Normal Control Subjects	Unknown	Johns Hopkins Medical Institute, Baltimore, Maryland, United States
	16	NCT03682458	Study of Neurodegenerative Diseases Induced Stem Cells in Patients and Healthy Family Controls.	Neurodegenerative Diseases	Unknown	Stefano Gambardella, Pozzilli, Isernia, Italy
	17	NCT03635294	Multi-Omics and IPSCs to Improve Diagnosis of Rare Intellectual Disabilities	Rare Intellectual Disabilities	Not Applicable	CHU Angers, Angers, France|HCL Lyon, Bron, France|CHU de Bourgogne, Dijon, France|CHU Nantes, Nantes, France|CHU Poitiers, Poitiers, France|CHU Rennes, Rennes, France
	18	NCT03181204	Modeling Bronchial Epithelium Modifications Associated With COPD Using iPS	Pulmonary Disease, Chronic Obstructive|Smoking	Unknown	Centre Hospitalier Universitaire de Montpellier, Montpellier, France
	19	NCT02772367	Generation of Heart Muscle Cells From Blood or Skin Cells of Breast Cancer Patients	Breast Cancer	Unknown	Memorial Sloan-Kettering Cancer Center, New York, New York, United States
	20	NCT00895271	Establishing Fibroblast-Derived Cell Lines From Skin Biopsies of Patients With Immunodeficiency or Immunodysregulation Disorders	Primary Immunodeficiency|DOCK8	Unknown	National Institutes of Health Clinical Center, 9000 Rockville Pike, Bethesda, Maryland, United States
Drug screening	21	NCT01943383	Pharmacogenomic Evaluation of Antihypertensive Responses in Induced Pluripotent Stem (iPS) Cells Study	Hypertension	Unknown	University of Florida, Gainesville, Florida, United States
	22	NCT04744532	iPSC-based Drug Repurposing for ALS Medicine (iDReAM) Study	Amyotrophic Lateral Sclerosis	Phase 1	Kyoto University, Kyoto, Japan|Kitasato University, Sagamihara, Japan|Tokushima University, Tokushima, Japan|Tottori University, Yonago, Japan
	23	NCT04097275	Induced Pluripotent Stem Cells for the Development of Novel Drug Therapies for Inborn Errors of Metabolism (iPSC-IEM)	Inborn Errors of Metabolism	Unknown	Children Hospital and Institute of Child Health, Lahore, Pakistan
	24	NCT03407040	Generation of Cancer Antigen-Specific T-cells From Human Induced Pluripotent Stem Cells (iPSC) for Research and Potential FutureTherapy	Gastrointestinal Cancers|Breast Cancer|Pancreatic Cancer|Melanoma|Lung Cancer	Unknown	National Institutes of Health Clinical Center, Bethesda, Maryland, United States
Therapy-based studies	25	NCT04945018	A Study of iPS Cell-derived Cardiomyocyte Spheroids (HS-001) in Patients With Heart Failure (LAPiS Study)	Heart Failure|Ischemic Heart Disease	Phase 1|Phase 2	St. Marianna University Hospital, Kawasaki, Japan|Nihon University Itabashi Hospital, Tokyo, Japan|The University of Tokyo Hospital, Tokyo, Japan|Tokyo Medical and Dental University Medical Hospital, Tokyo, Japan|Tokyo Metropolitan Geriatric Medical Center, Tokyo, Japan
	26	NCT03403699	Human iPSC for Repair of Vasodegenerative Vessels in Diabetic Retinopathy	Diabetes Complications|Diabetic Retinopathy	Unknown	University of Alabama at Birmingham, Birmingham, Alabama, United States
	27	NCT04696328	Clinical Trial of Human (Allogeneic) iPS Cell-derived Cardiomyocytes Sheet for Ischemic Cardiomyopathy	Myocardial Ischemia	Phase 1	Osaka University Hospital, Suita, Osaka, Japan
	28	NCT04339764	Autologous Transplantation of Induced Pluripotent Stem Cell-Derived Retinal Pigment Epithelium for Geographic Atrophy Associated With Age-Related Macular Degeneration	Age-Related Macular Degeneration	Phase 1|Phase 2	National Institutes of Health Clinical Center, Bethesda, Maryland, United States
	29	NCT04982081	Treating Congestive HF With hiPSC-CMs Through Endocardial Injection	Cardiovascular Diseases|Congestive Heart Failure|Dilated Cardiomyopathy	Phase 1	Help Therapeutics, Nanjing, Jiangsu, China
	30	NCT03971812	Organoids Derived From Induced-Pluripotent Stem Cells (iPS) From Patients With High Grade Astrocytoma	Glioma	Unknown	Assistance Publique HÃ´pitaux de Marseille, Marseille, France
	31	NCT03763136	Treating Heart Failure With hPSC-CMs	Heart Failure	Phase 1|Phase 2	HelpThera, Nanjing, Jiangsu, China
